# Innovation in patient-centered care: lessons from a qualitative study of innovative health care organizations in Washington State

**DOI:** 10.1186/1471-2296-13-120

**Published:** 2012-12-14

**Authors:** Peter Reed, Douglas A Conrad, Susan E Hernandez, Carolyn Watts, Miriam Marcus-Smith

**Affiliations:** 1 Pediatric Resident, University of Vermont, Burlington, VT, USA; 2 Department of Health Services, University of Washington, Box 357660, Seattle, WA, 98195-7660, USA; 3 Department of Health Administration, Virginia Commonwealth University, P.O. Box 980203, Richmond, VA, 23298-0203, USA; 4 Department of Health Services, University of Washington, Box 359455, Seattle, WA, 98105-9928, USA; 5 Department of Pediatrics, University of Vermont, Burlington, VT, 05401, USA

**Keywords:** Patient-centered care, Primary care, Health care reform

## Abstract

**Background:**

Growing interest in the promise of patient-centered care has led to numerous health care innovations, including the patient-centered medical home, shared decision-making, and payment reforms. How best to vet and adopt innovations is an open question. Washington State has been a leader in health care reform and is a rich laboratory for patient-centered innovations. We sought to understand the process of patient-centered care innovation undertaken by innovative health care organizations – from strategic planning to goal selection to implementation to maintenance.

**Methods:**

We conducted key-informant interviews with executives at five health plans, five provider organizations, and ten primary care clinics in Washington State. At least two readers of each interview transcript identified themes inductively; final themes were determined by consensus.

**Results:**

Innovation in patient-centered care was a strategic objective chosen by nearly every organization in this study. However, other goals were paramount: cost containment, quality improvement, and organization survival. Organizations commonly perceived effective chronic disease management and integrated health information technology as key elements for successful patient-centered care innovation. Inertia, resource deficits, fee-for-service payment, and regulatory limits on scope of practice were cited as barriers to innovation, while organization leadership, human capital, and adaptive culture facilitated innovation.

**Conclusions:**

Patient-centered care innovations reflected organizational perspectives: health plans emphasized cost-effectiveness while providers emphasized health care delivery processes. Health plans and providers shared many objectives, yet the two rarely collaborated to achieve them. The process of innovation is heavily dependent on organizational culture and leadership. Policymakers can improve the pace and quality of patient-centered innovation by setting targets and addressing conditions for innovation.

## Background

“Patient-centered care” is an oft-touted ideal in health care today, yet its interpretation varies considerably: [1] the Institute of Medicine report, *Crossing the Quality Chasm,* defines patient-centered care as clinical decision-making that flows from patient values; [2] the patient-centered medical home, a delivery mechanism of patient-centered care, encompasses a broad range of goals, including quality, safety, and payment reform, in addition to patient-centered clinical decision-making and “whole person” care [3]. Growing interest in patient-centered care coincides with growing attention to quality improvement and cost containment in the U.S. health care system [2,4-6]. This attention has spurred development of several vehicles to transform health care delivery, including new or renewed innovations such as payment reforms, accountable care organizations, shared decision-making, and the patient-centered medical home. Yet exactly how these innovations can best be vetted and adopted remains an open question.

Patient-centered care is one of several strategic goals pursued by health care organizations. Potential motivations to innovate in patient-centered care include policy mandates, available payment bonuses and research grant dollars, business opportunities, and moral imperative. But deterrents also exist, for example, the time and expense to adopt electronic health records and difficulty managing change in the relationships between payers and providers. To better understand health care organizations’ rationales for choosing -- or not -- to innovate in patient-centered care and their experiences of the process of innovation, we queried a range of innovative health plans, provider organizations, and primary care clinics in Washington State about their goals relating to patient-centered care, strategies used to achieve those goals, and challenges encountered along the way. We aimed to get an in-depth and personal perspective of patient-centered care innovation from the people at the front lines of health care delivery and innovation.

Washington State has been a leader in health care reform and a rich laboratory for patient-centered innovations. Legislation stemming from the state’s Blue Ribbon Commission for Health Care Reform 2007 report [7] reflects these innovations by promoting (1) the patient-centered medical home (PCMH), including the Washington Patient Centered Medical Home Collaborative [8]; (2) shared decision-making (SDM), including a Shared Decision-Making Demonstration project and legal protections for physicians who formally use shared decision-making tools [9]; and (3) payment reform, including the Patient Centered Medical Home Multipayer Reimbursement Model [8], which protects demonstration participants from antitrust action related to tight payor-provider collaboration. Private organizations in Washington are also leading reform efforts, exemplified by the Puget Sound Health Alliance “Community Checkup” reports [10] and Group Health Cooperative’s medical home model [11]. In addition to Washington State’s efforts, the federal Patient Protection and Affordable Care Act (PPACA) of 2010 promotes formation of accountable care organizations (ACOs) among its many reforms [12].

This paper’s view of patient-centered care innovation draws most directly from the concept of the patient-centered medical home [8], which highlights several dimensions:

• Comprehensive primary care

• Ready access to care

• Continuity of care

• Shared decision making between providers and patients

• Alignment of payment incentives with integrated care delivery

• Cultural competency of care

This study’s focus on four “pillars” of patient-centered care innovation (working toward a medical home, shared decision making, accountable care organization design, and payment reform) effectively captures each of those dimensions.

### Purpose of this paper

We hope to shed light on the experience of innovation in patient-centered care from the perspective of various health care organizations through directed interviews of key informants ranging from primary care providers to health system leaders to health insurance executives. Our study was restricted to organizations in the Puget Sound region of Washington State, yet we anticipate our findings will be useful across the United States precisely because (1) Washington is at the forefront of patient-centered care innovation and (2) the Puget Sound region contains – and our study includes – a great diversity health care delivery and financing organizations. Thus, common themes from across various organizations may be particularly robust when applied across the country. We are cognizant, however, that there may be a pervasive ethos specific to this region that could limit the generalizability of our findings.

Interviews were structured not to test specific hypotheses or to determine “best practices” for patient-centered care innovation, but rather to explore how each organization elaborated on the theoretical frameworks of organizational innovation developed by Nadler and Tushman, Nembhard et al., Cooke, Crossan, and Greenhalgh et al., which examine implementation and management of change and distinguishing features of innovation [13-17]. These include (1) goals, (2) drivers – or underlying motivations – of those goals, (3) strategies and activities employed to achieve the goals, (4) barriers and facilitators, (5) perceptions of the process.

## Methods

We conducted semi-structured key-informant interviews lasting 45 to 60 minutes each with five health plans (health insurance providers), five provider organizations, and ten clinics (two from each provider organization). Health plans were a state-sponsored health plan; a public purchaser; a private, for-profit health plan; and two private, not-for-profit plans. Provider organizations included a multispecialty, physician-owned group; an independent practice association; and three not-for-profit, multihospital, multispecialty groups.

Our primary goal was to capture the process of innovation as it unfolds within an organization. Therefore, we chose to study organizations actively participating in at least one of the following innovation projects: Centers of Medicare & Medicaid Services Group Practice Demonstration, Washington State Shared Decision Making Demonstration, Washington State Patient Centered Medical Home Multi-Payer Reimbursement Model and Washington State Patient Centered Medical Home Collaborative, forming an ACO, or creating a patient-centered medical home.

Interviews were conducted in person or by phone with one interviewee and two research team members. Interviews were recorded and transcribed by a third-party, professional transcription service. Transcriptions were spot-checked for accuracy against notes taken during the interviews.

Limited resources necessitated choosing key informants rather than drawing from a large sample within each organization, so we selected individuals whom we expected would be most involved with innovation at a strategic level and/or were implementing innovations on the ground. At each health plan and provider organization, we interviewed a senior medical executive and a senior operations executive (10 health plan interviews; 10 provider organization interviews). Within each provider organization, we conducted interviews at an “innovator” clinic site and a “comparison” site as determined by the organization's executives. Designation as an “innovator” was based on being more advanced in implementing one or more patient-centered care innovations at the center of this study (PCMH, SDM, payment reform, and/or ACO development). At each site, we interviewed the medical director, quality improvement leader, practice manager, and where possible, a primary care provider without an administrative role (33 practice site interviews).

At least two members of the research team read each interview transcript and generated a list of themes. These two (or more) lists were not verified against each other. Rather, all themes from each reading, divided by class of organization, were sorted and consolidated by consensus of the five-member research team through an iterative process of discussions. (For example, one member of the team would propose that theme A and theme B could sensibly be combined into a theme C. The team would discuss this possibility and either choose to consolidate the themes or leave them separate. Later, theme D might be considered for consolidation with theme C. Finally, themes were organized to conform with the frameworks of innovation – goals, drivers, strategies, barriers and facilitators, and perceptions.)

This research was reviewed and deemed exempt by the Human Subjects Division of the University of Washington. Informed consent was obtained from all interviewees. This research conforms to the RATS guidelines on qualitative research.

## Results

### Goals (Table 1)

**Table 1:** Strategic Goals

	**Health Plans**		**Provider Organizations**		**Clinics**	
**Goals**	Public Plan or Purchaser	Private Plans	Multispecialty Groups	Independent Practice Association	Innovator Site	Comparison Site
Survive	X	X	X	X	X	X
Improve quality	X	X	X	X	X	X
Contain costs	X	X	X			
Deliver appropriate care	X	X				
Improve patient experience/satisfaction			X	X	X	X
Manage chronic disease			X	X	X	X
Respond effectively to PPACA	X	X				
Comply with PPACA	X	X				
Build strategic partnerships			X			
Position organization for new competitive environment			X			
Increase market share			X			
Achieve high provider/staff satisfaction					X	X
Engage and develop employees					X	X
Improve access					X	X
Enhance care coordination					X	X
Create a medical home					X	

Health plans, provider organizations, and clinics shared the goals of quality improvement and organizational survival. Health plans and provider organizations were both interested in cost containment, but this goal did not emerge from providers. One health plan executive summarized, “Our goals, as always, are to manage the cost of care, improve the quality of care, and create insurance products that people want to buy.” Only health plans were concerned with responding to PPACA at the level of a strategic goal: “A major strategic goal or area of strategic emphasis is our response to health reform.” While not a strategic goal, provider organizations and individual providers were preparing for changes resulting from PPACA.

In addition to quality improvement and cost containment, provider organizations emphasized expanding their market power, including increasing market share and building strategic partnerships. “We’re very focused on developing strategic relationships with other organizations,” said one provider organization executive. The independent practice association was planning to create an ACO.

Patient-oriented goals other than general quality improvement, such as improving patient satisfaction and chronic disease management, were shared by provider organizations and their clinics. Health plans tended not to share these goals and were more interested in lowering cost of care. Practice sites were especially engaged in staff and provider satisfaction efforts. Three out of five innovator sites were experimenting with medical homes.

### Drivers

We asked health plans and clinics what motivated their choices of goals. Both sets of organizations cited PPACA as a strong driver for choosing their objectives. The clinics’ choices of goals were influenced by their parent provider organizations and the demands of patient-consumers. Rising health care cost was a concern of the private health plans. As one health plan executive put it, “We’re falling off a fiscal cliff because of the cost of health care.” Common drivers were not apparent among provider organizations.

### Strategies and activities (Table 2)

**Table 2:** Strategies and Activities

	**Health Plans**		**Provider Organizations**		**Clinics**	
**Strategies and Activities**	Public Plan or Purchaser	Private Plans	Multispecialty Groups	Independent Practice Association	Innovator Site	Comparison Site
**Information Technology and Management**						
Make greater and better use of data	X	X	X	X	X	X
Use electronic medical records					X	X
Use e-prescribing					X	X
**External Environment and Relationships**						
Change/influence PPACA regulations		X				
Account for PPACA in planning			X			
Transform organization of health care delivery	X	X				
Change reimbursement away from fee-for-service	X	X				
Tailor payment to shape delivery system		X				
Cultivate strategic partnerships			X			
Prepare to become an ACO			X			
**Patient-Centeredness and Product Design**						
Improve customer service			X			
Engage patients in their health/health care		X	X	X		
Survey patient experience/satisfaction			X		X	X
Improve appropriateness of care	X	X	X			
Improve care coordination			X	X	X	X
Develop value-based benefits		X				
Focus on a specific population	X				X	X
eVisits					X	X
Experiment with medical home	X	X	X		X	
**Work Processes and Tasks**						
Use strategic framework for improvement			X			
Centralize some tasks					X	X
Advanced scheduling					X	X
Expand nurse and mid-level providers' scope of practice			X		X	X
RNs lead chronic disease management					X	
Use scribes					X	X
**Human Resources**						
Bolster human resource efforts			X	X		
Recruit providers who are a good cultural fit			X			
Recruit within the organization			X			
Engage employees			X		X	X
Train physician-leaders			X			
Financial rewards for performance improvement			X			

The strategies employed by different organizations, including those being implemented actively and those that were planned, reflected their respective missions and spheres of influence, though there were a few strategies that cut across all types of organization. In many cases the line between “goals” and “strategies” is blurry. We attributed themes as strategies when they were described in support of pursuing a goal (e.g., creating an ACO was a goal of the IPA, but for some other provider organizations it was a strategy in support of cost and quality goals).

Health plan-only strategies included influencing PPACA regulations, restructuring provider payment, redesigning benefits, and completely transforming the organization of health care delivery. Some public plans were interested in tailoring services to their specific membership (e.g., Medicaid plans were focused on young mothers and children).

Provider organizations had internal strategies such as improving quality and efficiency through workflow changes, using health information technology, and developing human capital, although they also were planning for PPACA implementation, particularly building the foundation for ACOs. Compared to health plans and the comparison site clinics, provider organizations and innovator clinics were more interested in employing patient-oriented strategies in support of improved customer service and patient satisfaction goals. Most clinics were trying to improve their work processes but were not much engaged in human resource development.

The only common strategy across the three levels -- health plan, provider organization, and practice site (clinic) -- was to make better use of available data within each organization. For example, health plans wanted to pull more information from their claims data, and provider organizations, and their clinics wanted to use patient satisfaction data and quality metrics to improve physician performance.

### Barriers and facilitators to achieving goals (Table 3)

**Table 3:** Barriers and Facilitators

	**Health Plans**		**Provider Organizations**	
**Barriers**	Public Plans or Purchasers	Private Plans	Multispecialty Groups	Independent Practice Association
Insufficient analytic power	X	X		
Regulatory restrictions	X	X		
Economic constraints	X			
Insufficient creativity		X		
Pay for production		X	X	
Insufficient financial capital	X		X	X
External Inertia	X	X	X	X
Internal Inertia	X	X	X	X
Insufficient information technology			X	
Insufficient human capital			X	X
**Facilitators**				
Data resources	X	X		
Size	X	X		
Experience		X		
PPACA		X		
Leadership	X	X	X	
Personnel			X	X
Agility/responsiveness			X	X
Culture of continuous improvement			X	
Willingness to take risks			X	X

* No clear barriers or facilitators emerged from the clinic site interviews.

We asked every organization what factors facilitated or inhibited the pursuit of their goals. Significant themes emerged only from health plans and provider organizations. Health plans and provider organizations cited inertia or resistance to change, both within the organization and in the external environment, as a principal barrier to innovation. Other impediments to innovation were deficits of resources, including human capital, financial capital, information technology, and creativity. Public health plans were feeling greater financial pressures than private plans, reflecting Washington State’s fiscal crisis. Health plans found regulations, particularly state law that restricts providers from bearing financial risk without meeting stringent financial and administrative requirements [18], to be limiting, while pay-for-production restricted both health plans and provider organizations because it precluded creativity with payment reform.

“The biggest impediment is the fact that people are on … the hamster wheel…. The current financing system we have in health care demands that they remain on the hamster wheel. So I’m trying to ask them to say, ‘Let’s slow [it] down long enough to think about whether there could be a different future.’” – a provider organization executive.

The Affordable Care Act was seen by private health plans as a facilitator for innovation because it will apply pressure to patients, employers, and providers, in addition to insurance companies, and may encourage cooperation among these stakeholders. Despite concerns about resource deficits, all organizations lauded their respective strengths, including leadership, agility, data resources, and commitment of their personnel. Provider organizations also cited cultural elements of continuous improvement and willingness to try new things as facilitating factors.

It is notable that different types of health care organizations exist in silos created by varying mission and regulations; these divisions were apparent in discussion of barriers and facilitators. Each organization tackled emergent difficulties independently of other organizations, rather than by working with each other to overcome common challenges. For example, provider organizations cited flexibility and willingness to take risks as strengths, but they had not joined with health plans in overhauling pay-for-production, which both groups saw as a barrier.

### Perceptions

Despite private health plans’ perceptions that PPACA would facilitate innovation, most health plan executives had strong and negative, feelings about the health reform law, including uncertainty about what is in the bill and how regulations would be written. They were also concerned that PPACA would put increased strain on the health care system and raise costs. Health plans were cautiously optimistic about the promise of medical homes and largely skeptical of the value of shared decision-making.

Provider organizations were contemplating how their organizations could continue to compete against other, sometimes larger, provider organizations in a financially constrained and increasingly regulated health care market in the future. Executives of health plans and provider organizations agreed that fee-for-service payment does not work and must be reformed. They suggested alternatives such as payment for quality and for cost containment.

## Discussion

This is a qualitative study of innovation in a region incubating a variety of patient-centered innovations. Participating organizations were chosen because they were implementing or preparing to implement one or more patient-centered innovations, and because they represented a broad range of health care organizations. Patient-centered innovations were not often specified as strategic goals *per se*. However, organizations’ top priorities, such as survival, cost containment, and quality improvement, were consistent with patient-centered care. Reflecting the variety of organizational structures, each organization was unique in its set of goals and strategies despite a degree of shared interest in aligning incentives and developing ACOs.

We attempted to represent faithfully the ideas presented by the interviewees. For example, while managing chronic disease is an aspect of appropriate care delivery, we did not assume that provider organizations shared the goal of appropriate care delivery with health plans because it did not emerge explicitly in the interviews. This methodology may, on the surface, divide themes that actually overlap. It is important to keep in mind the missions of the organizations to which themes are attributed.

We anticipated that organizations with different structures -- the independent practice association versus the multispecialty groups, or public versus private health plans -- would have different methods and approaches to patient-centered innovation, and this was the case. For example, executives at the provider organizations described many similar strategic objectives and visions, but the primary care practices affiliated with those organizations varied in their alignment with the parent provider organization’s vision. For example, the independent practice association providers rarely discussed the IPA leadership’s goals or strategic initiatives, while the multispecialty group providers referred to the leaders’ goals repeatedly – suggesting a greater degree of integration between affiliated practices in the multispecialty groups, as compared to the IPA.

Goal selection was influenced by particular organizational leaders, the culture of the organizations, organizational structure, and especially external drivers. This is a dynamic time for patient-centered innovation in Washington State, with the rollout of PPACA underway, multiple ongoing state-sponsored demonstration projects, a fragile economic recovery, and severe, continuing state budget constraints. The organizations’ goals were a balance between ongoing mission-critical objectives, like quality improvement and organizational survival, and adaptations to the current fiscal, competitive, and regulatory environment, such as transitioning to PPACA implementation.

**Figure 1:**
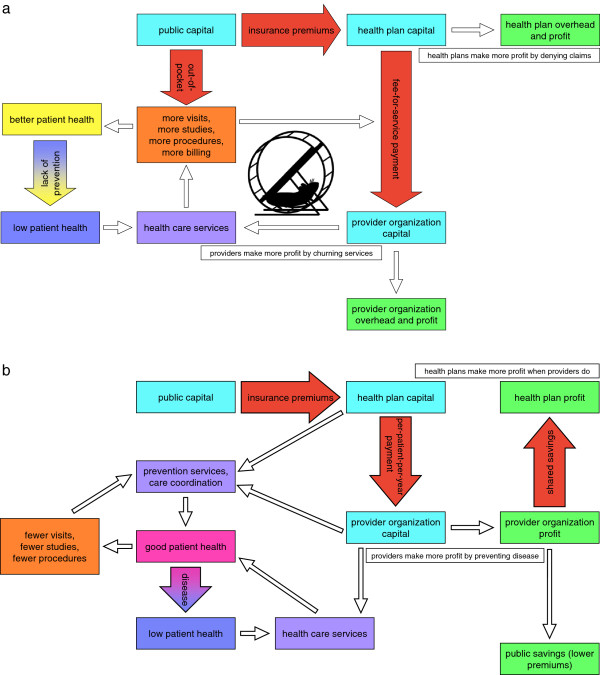
A comparison of current fee-for-service payment system (a) with a theoretical accountable care organization health system structure (b).

Across health care organizations, a principal barrier to successful innovation was fee-for-service, or “pay-for-production,” provider compensation as well as the misalignment of financial incentives between health plans, providers, and patients. Organizations struggled to find material, financial, intellectual, and creative space for patient-centered innovation within the fee-for-service system – the “hamster wheel.” Hallmarks of patient-centered care are prevention and proactive outreach. These are absent from the current organizational-financial model (Figure 1a), but they are central to a sound, patient-centered delivery model, perhaps accomplished through ACOs (Figure 1b). Both health plans and provider organizations were willing and eager to try new payment and risk-bearing systems, but they will need financial support and legal leeway to test these delivery and reimbursement models.

### Policy implications

The principal challenge in crafting policy is to find where organizations would not go on their own but could be coaxed or directed to do so for the public good with the right mix of cost-effective incentives and regulations. This study has helped to illuminate the factors that influence innovation, including leadership, organizational culture, and external drivers. Importantly, though, we found that organizations employed strategies and faced challenges that reflected their place in the health care market. For example, primary care clinics used work process changes rather than payment and benefit redesign, which were used by health plans, to enhance chronic disease management and quality improvement; health plans felt restricted by regulations while provider organizations cited human capital deficits as barriers.

The correlation between an organization’s ability to change an aspect of health care delivery and that organization’s choices of goals and strategies was expected, but striking nonetheless. With the exception of ACOs, the primary innovations happening in Washington State reside within each organization’s respective place in the spectrum of health care delivery and financing: shared decision-making and patient-centered medical home innovations are fundamentally at the provider level, while payment reform starts with regulations and health plans without requiring much provider input. Given that the organizations in our study are largely thinking about goals and strategies within their spheres of influence, it will take some out-of-the-box planning and/or higher-level leadership to make ACOs or comparable reforms possible.

The connection between an organization’s capacity and its chosen strategies and anticipated challenges also means that intra-organizational patient-centered innovations will either have to match health care organizations’ capabilities, or that organizations’ capacities must be bolstered, or both. In the coming new age of ACOs and integrated health care payment and delivery, a golden opportunity exists for each organization to make the best use of its capabilities and to partner with organizations that can make up for its deficits.

Concordantly, basic organization cultural and business norms, such as proprietary protections, will have to adapt in order for organizations to form effective ACOs, which rely on transparency and coordination between organizations. The shift from misaligned incentives under fee-for-service (Figure 1a) to aligned incentives of ACOs (Figure 1b) will aid in changing cultural norms of health care organizations, and we can glean some encouragement from providers’ rapid response to Medicare’s prospective payment (Diagnosis Related Groups) rollout in the 1980s [19-21]. Still, we can expect the transition from competitive to cooperative cultures – as distinct from behaviors – to be slow, and the specific path to forming these cooperative networks is challenging to conceive [22].

### Limitations and future research directions

By design, this qualitative study centered on a “deep dive” within a small, purposive sample of organizations (health plans, provider organizations, and their affiliated practices) in a particular state context. While this focus in a specific environment and selected organizations generated rich learning and a unique “proof of concept” of patient-centered innovation, the enhanced internal validity and insights from this approach must be weighed against potential limitations in generalizability to care settings in other contexts. Future research should apply the constructs of this extended qualitative study in large sample, multiple setting, and quantitative studies of patient-centered care.

## Conclusion

Patient-centered care innovations reflected distinct organizational perspectives. Health plans emphasized cost-effectiveness, while providers emphasized health care delivery processes. Health plans and providers shared many objectives, yet the two groups rarely collaborated to achieve them. The process of innovation is heavily dependent on organizational culture and leadership.

Policymakers can improve the pace and quality of patient-centered innovation by clearly establishing a vision of patient-centered care and by addressing the conditions for innovation: leadership development (training); smart regulation (incentives and leeway for experimentation); and inter-organizational cooperation (innovation in payment, regulation, and information sharing). Health care organizations are primed for major changes in health care financing and delivery; successful transformation of the health care system will require participation of and cooperation between providers, health plans, and policymakers.

## Abbreviations

PPACA: Patient Protection and Affordable Care Act; ACO: Accountable care organization.

## Competing interests

Authors have no competing interests, financial or non-financial, to declare.

## Authors’ contributions

PR participated in the design of the key informant interview instruments, conducted interviews, participated in the analysis, and was the lead author of the manuscript. DC developed the original study design, participated in the design of the key informant interview instruments, conducted interviews, participated in the analysis, and contributed to the writing of the manuscript. SH participated in the design of the key informant interview instruments, conducted interviews, participated in the analysis, and contributed to the writing of the manuscript. CW participated in the study design, participated in the design of the key informant interview instruments, participated in the analysis, and contributed to the critical revisions of the manuscript. MMS participated in the design of the key informant interview instruments, conducted interviews, participated in the analysis, and contributed to the writing of the manuscript. All authors read and approved the final manuscript.

## Pre-publication history

The pre-publication history for this paper can be accessed here:

http://www.biomedcentral.com/1471-2296/13/120/prepub
